# Carbon Dynamics within Cyclonic Eddies: Insights from a Biomarker Study

**DOI:** 10.1371/journal.pone.0082447

**Published:** 2013-12-30

**Authors:** Iván J. Alonso-González, Javier Arístegui, Cindy Lee, Anna Sanchez-Vidal, Antoni Calafat, Joan Fabrés, Pablo Sangrá, Evan Mason

**Affiliations:** 1 Instituto de Oceanografía y Cambio Global (IOCAG), Universidad de Las Palmas de Gran Canaria, Las Palmas de Gran Canaria, Spain; 2 Spanish Bank of Algae (BEA), Telde, Spain; 3 School of Marine and Atmospheric Sciences, Stony Brook University, Stony Brook, New York, United States of America; 4 GRC Geociències Marines, Facultat de Geologia, Universitat de Barcelona, Barcelona, Spain; 5 UNEP Shelf Programme Facility, UNEP/GRID-Arendal, Arendal, Norway; 6 Grup d'Oceanografia Fisica, Institut de Ciències del Mar, CMIMA-CSIC, Barcelona, Spain; 7 Mediterranean Institute for Advanced Studies (IMEDEA), Esporles, Spain; Mount Allison University, Canada

## Abstract

It is generally assumed that episodic nutrient pulses by cyclonic eddies into surface waters support a significant fraction of the primary production in subtropical low-nutrient environments in the northern hemisphere. However, contradictory results related to the influence of eddies on particulate organic carbon (POC) export have been reported. As a step toward understanding the complex mechanisms that control export of material within eddies, we present here results from a sediment trap mooring deployed within the path of cyclonic eddies generated near the Canary Islands over a 1.5-year period. We find that, during summer and autumn (when surface stratification is stronger, eddies are more intense, and a relative enrichment in CaCO_3_ forming organisms occurs), POC export to the deep ocean was 2–4 times higher than observed for the rest of the year. On the contrary, during winter and spring (when mixing is strongest and the seasonal phytoplankton bloom occurs), no significant enhancement of POC export associated with eddies was observed. Our biomarker results suggest that a large fraction of the material exported from surface waters during the late-winter bloom is either recycled in the mesopelagic zone or bypassed by migrant zooplankton to the deep scattering layer, where it would disaggregate to smaller particles or be excreted as dissolved organic carbon. Cyclonic eddies, however, would enhance carbon export below 1000 m depth during the summer stratification period, when eddies are more intense and frequent, highlighting the important role of eddies and their different biological communities on the regional carbon cycle.

## Introduction

Understanding the mechanisms that control carbon export to the deep ocean is a major outstanding concern in oceanography. Sinking particulate organic carbon (POC) fluxes measured with current techniques (sediment traps and thorium approaches) are not consistent with the oxygen utilization rates measured in the deep ocean [1], [2]. This apparent imbalance indicates either the existence of unknown sources of organic carbon, an overestimation of the metabolic activity in the dark ocean, or an underestimation of the vertical particle flux.

One possible mechanism to supply some of the “missing carbon” locally would be intermittent and undersampled carbon pulses by mesoscale eddies. Nevertheless, model results and field studies that address the effects of eddies on organic matter fluxes have shown conflicting results [3]. A limited number of studies have shown direct evidence of enhanced carbon export mediated by mesoscale eddies [4]–[6]. However, recent interdisciplinary programs that focused on the effects of eddies on carbon export (E-Flux in the North Pacific and EDDIES in the North Atlantic) have shown different results. Surprisingly, both programs concluded that the studied eddies did not enhance carbon flux, although they increased the flux of biogenic silica [7], [8]. More recently, a study conducted in the Canary Current region reported new results that further fuel this controversy [9]. These authors found that the eddy field generated south of the Canary Islands more than doubled the POC export below the mixed layer compared to stations outside the influence of the eddy field. Data from that work were obtained from free floating sediment trap deployments during a short period characterized by warm and stratified waters, but also intense winds that enhanced eddy development by Ekman pumping. Indeed, one hypothesis proposed by the E-Flux and EDDIES programs was that intermittent carbon pulses might be undersampled during research cruises. Determining the influence of eddies on carbon export and organic matter composition using time series observations could be useful to test this hypothesis.

Here, with the aim of addressing this challenge, we measured POC, amino acid and chloropigment fluxes and compositions in samples collected from a mooring deployed in the area of generation of cyclonic eddies south of Gran Canaria (Canary Islands). The island sheds oceanic eddies all year round [10]–[12], making this region an ideal site for the investigation of the effects of newly formed eddies on the biogeochemistry of an oligotrophic subtropical system. This study reports results from the longest time series to date of organic matter composition and export within a cyclonic eddy field.

## Materials and Methods

### Study area and sampling design

**Figure 1:**
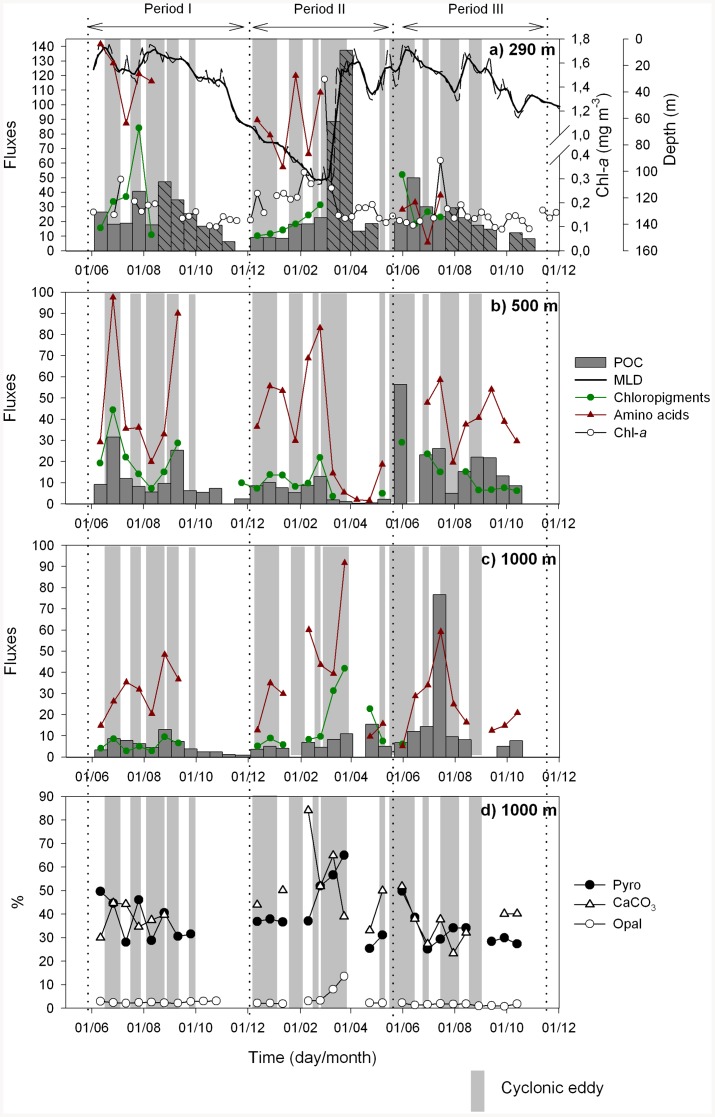
Impact of the cyclonic eddy field on organic matter fluxes and composition. POC (dark grey bars; mg m^−2^ d^−1^), amino acids (red triangles; µmol m^−2^ d^−1^) and chloropigment (green dots; µg m^−2^ d^−1^) fluxes collected with PPS3 sediment traps at a) 290 m, b) 500 m and c) 1000 m. Dark grey shaded bars indicate POC fluxes derived from an Indented Rotating Sphere Carousel (IRSC) sediment trap located 30 m above (260 m) of the PPS3 [50]. Light grey bars indicate “eddy” conditions, white spaces indicate “non-eddy” conditions [see 12]. The thicker and thin black lines on the upper panel represent the seasonal variability of the mixed layer depth measured each 3 days and smoothing with a 15-day moving average, respectively; white dots stand for the surface chlorophyll derived from satellite images [see 50 for more details]. d) Pyropheophorbide mole% (mesozooplankton indicator), % CaCO_3_ and % biogenic opal measured in the 1000 m samples. Period I: June 2005 to December 2005, Period II: December 2005 to May 2006, and Period III: May 2006 to December 2006. POC flux bar missed means no measurement exists.

A sediment trap mooring was deployed at 27°29′57″N; 16°15′19″W, 3600 m bottom depth, for three 6-month periods (from June 2005 to December 2006). Periods I, II, and III are shown in Fig. 1. Rough sea conditions forced the mooring location to be situated closer to the islands during Period III (27°30′4″N; 15°44′32″W, 2500 m bottom depth). Since the mooring line was placed in Spanish waters deeper than 3000 m and not involved endangered or protected species, no specific permission was necessary. The mooring accommodated 3 PPS3/3 sediment traps (TECHNICAP) at 290, 500 and 1000 m; all cups were poisoned with mercuric chloride, and samples were processed according to the protocol described in detail by Heussner et al., [13]. Aanderaa RCM7/8 current meters were placed on the mooring 2 m below each sediment trap. The presence of eddies was monitored by combining current-meter temperature anomalies with sea surface temperature (SST) and chlorophyll from satellite images. Negative temperature anomalies from the mooring that were associated with cyclones, matched well with SST negative anomalies obtained from satellite images [12].

Sediment traps with a collection area of 0.125 m^2^ were programmed to collect particles in a time-series mode with a sampling interval of 15 days; this interval was selected based on the initial diameter of cyclonic eddies (50–70 Km) and their advection velocities (3–4 Km day^−1^) [10], [14]. According to this, an eddy would take 15 to 20 days to cross the mooring, which is close to the time resolution of the sediment trap sampling (15 days). This estimated period for an eddy's passage over the mooring site is consistent with the time period of the corresponding negative temperature anomalies and SST eddy signals that were recorded in the mooring area, which was 15 days on average [12]. This results in a mean residence time of the eddies at the mooring site of about 15 days, independent of whether the site is crossed by the center or the periphery of the eddy.

We are aware that small particles with very low sedimentation rates (e.g. <5 m day-1) may be collected by deeper traps, and may have originated from more distant sources. However, using laboratory and model studies of flow perturbation by obstacles (like Gran Canaria) Jimenez et al., [11] show that most of the water in the wake region related to eddies comes from the recirculation zone located just downstream of the obstacle. Hence, during eddy events the collection area most likely will be rather small and located in the recirculation region just downstream of the island. Thus, funnel effects are not likely to be crucial during eddy-periods; indeed, this can be clearly seen in color images of the Gran Canaria area [15].

Taking into account sedimentation rates of particles and shedding velocities of eddies, we can assume that there is an overlap in the effects of consecutive eddies that are shed with less than a 10-day gap. Therefore, it is not feasible to ascribe the material collected in the sediment traps to a single eddy, but to the cyclonic eddy field as a whole. To study the differential effects of the cyclonic eddy field on carbon fluxes and transfer efficiency within each period, we distinguished between two distinct dynamical regimes: “eddy” and “non–eddy” conditions. We consider “eddy” conditions to be the time interval that contains the bulk of eddy events (e.g., from July to September in Period I), and vice versa for “non-eddy” conditions. Under “eddy” conditions, certain time frames may be observed with no eddies present, but they were under 10 days in length. This finding justifies our approach of clustering and averaging samples as a function of “eddy” and “non-eddy” conditions.

### POC and biomarker analysis

Particulate organic carbon (POC), amino acids and chloropigments were measured as described earlier [9]. Organic carbon analyses were performed with a Perkin–Elmer 2400 CHN elemental analyzer [16]. DOC adsorption on GF/F filters (<4% of the POC signal) was subtracted from samples to avoid overestimation of POC [17].

Chloropigment concentrations (chlorophyll *a*, pheophytin *a*, pheophorbide *a*, and pyropheophorbide *a*) were determined in solvent extracts of filtered samples using reverse-phase High Performance Liquid Chromatography (HPLC) as described in detail by Lee et al., [18] and Wakeham et al., [19].

Amino acids were measured by HPLC on the same filters analyzed for pigments, using pre-column *o*-pthaldialdehyde (OPA) derivatization after hydrolysis [18], [19]. In addition, we calculated the degradation state of organic matter in each sample using an amino-acid-based Degradation Index [20], [21].

### Principal component analysis (PCA)

Principal component analysis (PCA) was used here to quantitatively assess variation in the organic composition of sinking particles that were collected during eddy vs. non-eddy periods at 290, 500 and 1000 m depth in the Canary Current region. PCA is commonly used in the analysis of complex organic gechemical datasets [22]–[24]. We applied PCA to a dataset that included both pigment and total hydrolyzed amino acid (THAA) compositions. Prior to performing the analysis, the mole% values of individual THAA and pigment compounds in each sample were normalized by subtracting the mean of all values and dividing by the standard deviation of all values for each class separately [20], [25]. All PCAs for this study were carried out on Sirius for Windows™ Pattern Recognition System (version 7.0).

### Cyclonic eddy trajectories from a numerical model

The trajectories of simulated cyclonic eddies at the Canary Islands were obtained by applying an eddy tracking algorithm to surface velocity outputs from a 50-year climatological ROMS (Regional Ocean Modeling System; [26]) solution of the Canary Basin. Eddies were tracked over a 40-year period. The eddy tracker is based on the Okubo-Weiss parameter, and follows a methodology that has previously been applied to the tracking of mesoscale eddies observed by altimetry [27], [28]. A full description of the eddy tracking method is given by Mason et al., [28]. The ROMS solution is fully eddy resolving with a horizontal resolution of 7.5 km, and has been validated by Mason et al., [28] and Mason et al., [29]. The seasonal cycle of the model eddy kinetic energy (EKE) over the Canary archipelago compares well with observations computed using satellite altimeter sea surface height, indicating that the model is a reliable predictor of eddy activity. We preferred to use model eddy trajectories rather than observed trajectories such as from the Chelton et al., [30] database because of the proximity of the mooring to land, that renders altimeter data to be unreliable. Nevertheless, we note that Mason et al., [31] estimate from altimeter data (1992 to 2012) that approximately 10 eddies per year pass through the lee region, in good agreement with the results of Piedeleu et al., [12] who reported 10 cyclones per year at the mooring.

## Results and Discussion

### Impact of cyclonic eddies and zooplankton activity on organic matter fluxes and composition

The Eulerian measurements recorded at the fixed position of our study site allowed us to evaluate the impact of cyclonic eddies on the local biogeochemistry. An average of 10 cyclonic eddies per year were identified (light grey bars on Fig. 1). Specifically, during Periods I and III (summer and autumn), 5 and 4 eddy events, respectively, were observed in summer, coinciding with relatively higher intensities of the incident flow and wind shear. In Period II (winter and spring), 4 eddies were generated in winter and 1 in spring [12].

Figure 1 illustrates the temporal evolution of POC, chloropigment and amino acid fluxes as well as “eddy” (group of light grey columns) and “non-eddy” (long white spaces) conditions. Our results reveal a significant influence of cyclonic eddies on POC fluxes during summer and autumn periods (Periods I and III), when surface waters are stratified and eddies are more intense due to the combined effect of flow perturbation and wind forcing [11], [32], [33]. In these periods, average carbon export during eddy conditions was approximately 2 to 4 times higher than that measured during non-eddy conditions (Table 1). However, during Period II (winter and spring), cyclonic eddies seem to have little effect on POC export compared to non-eddy conditions (Table 1, Fig. 1).

**Table 1:** Influence of the cyclonic eddy field on POC fluxes.

	Period I			Period II (bloom period)			Period III		
Depth (m)	eddy	non-eddy	R _eddy/non-eddy_	eddy	non-eddy	R _eddy/non-eddy_	eddy	non-eddy	R _eddy/non-eddy_
290	29.1 (9.1)	16.4 (7.8)	1.8 (p<0.05)	14.3 (6.2)	64.5 (59.4)	0.2 (p>0.05)	29.2 (10.9)	14.9 (2.6)	2.0 (p<0.05)
**T_eff_** (500/290 m)	49.9%	32.9%		48.9%	1.6%*		84.2%	66.4%	
500	14.5 (9.8)	5.4 (2.2)	2.7 (p<0.05)	7.0 (2.6)	1.0 (0.9)	7.0* (p<0.05)	24.6 (17.3)	9.9 (3.2)	2.5 (p<0.05)
**T_eff_** (1000/500 m)	54.4%	38.9%		90%	100%*		86.6%	61.6%	
1000	9.9 (2.9)	2.1 (1.1)	3.8 (p<0.01)	6.3 (2.7)	5.1 (1.2)	1.2 (p>0.05)	21.3 (27.2)	4.1 (1.9)	3.5 (p<0.05)

Average (±1 SD) fluxes (mg m^−2^ d^−1^) of POC for “eddy” and “non-eddy” conditions. T_eff_ = mesopelagic transfer efficiency defined as 500/290 m and 1000/500 m POC flux. R _eddy/non-eddy_ = POC flux ratio between eddy and non-eddy conditions. Average eddy-induced carbon flux increase at 1000 m calculated as POC fluxes during “eddy” conditions minus POC fluxes during “non-eddy” conditions (9.9+21.3)/2−(2.1+4.1)/2) = 12.5 mg C m^−2^ d^−1^. I: June 2005–December 2005; II: December 2005–June 2006; III: June 2006–December 2006.

Anomalous values mediated by vertically migrating zooplankton (see text and Fig. 3 for explanation).

In periods I and III, total chloropigment fluxes during eddy conditions were also 2 to 4 times higher than during non-eddy conditions (Fig. 1). Since pigments are originally derived from surface phytoplankton, we hypothesize that cyclonic eddies enhanced both primary production and POC fluxes in this region. Indeed, previous studies have demonstrated that cyclonic eddies (particularly those in their early stages of formation, close to the islands) may increase by several times the chlorophyll concentration and primary production with respect to ambient waters [15], [33]–[35]. Total amino acid fluxes at 500 and 1000 m were up to an order of magnitude higher within cyclonic eddies relative to non-eddy conditions (Figs. 1b,c). Similar to the chloropigments, higher amino acid fluxes within cyclonic eddies relative to non-eddy conditions are likely to be a result of enhanced primary production caused by nutrient pumping. We hypothesize that cyclonic eddies generated during winter/spring (Period II) did not have a significant effect on primary production and carbon export because the surface waters were already mixed down to 120 m (Fig. 1a) and thus nutrient enriched, in agreement with recent observations [3], [36]. Another possible explanation for the higher fluxes during the stratification period is that summer-autumn eddies may entrain enriched POC waters from other far-field regions like the eutrophic NW Africa upwelling system. However, since pigments and amino acids are highly labile compounds, this hypothesis is less likely.

**Figure 2:**
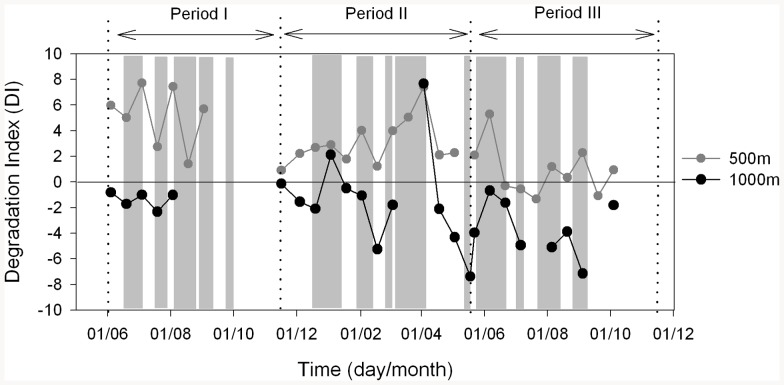
Organic matter degradation state. Time evolution of the amino acid Degradation Index (DI) of the organic matter collected at 500 m (grey circles) and 1000 m (black circles) depth. See Figure 1 for period dates.

Alternatively, the higher POC, chloropigment and amino acid fluxes measured during eddy conditions could originate from lower degradation rates of these components relative to those during non-eddy conditions. In this case, a lower degradation state of the collected organic matter would be expected. Figure 2 shows the degradation state of the organic matter collected at 500 and 1000 m using an amino-acid-based Degradation Index (DI) [20], [21]. Since the amino acid composition among marine organisms is so similar, the variation in amino acid composition arises primarily from degradation [20], [21]. The more negative the DI, the more degraded the organic matter in the sample, while positive DI values suggest fresher organic mater. Both 500 and 1000 m DI values show a lesser degradation state for the organic matter collected during eddy relative to non-eddy conditions (Fig. 2). This result is more consistent with a slower POC flux attenuation with depth within cyclonic eddies than that during non-eddy conditions. The degree of flux attenuation can be expressed as the ratio of POC flux between two depth levels (transfer efficiency, T_eff_). During eddy conditions POC T_eff_ between 500/290 m and 1000/500 m ranged from 49 to 90%, whereas for non-eddy conditions POC T_eff_ ranged from 2 to 66% (Table 1). This pattern of more efficient POC transfer within eddies must be related to the biogeochemical perturbations generated by these mesoscale features (e.g, higher phytoplankton cell size, higher particle sinking velocities or higher heterotrophic activity in non-eddy conditions). However, since trapping efficiency can be lower at mesopelagic depths [37], [38], and an averaging approach was used in Table 1, these transfer efficiency calculations must be taken with some degree of caution.

Overall, these findings suggest that the cyclonic eddy field generated south of the Canary Islands acts as a physical-biological pump of fresh organic matter to the deep ocean. Thus, our observations contrast with results obtained in cyclonic eddies in the lee of Hawaii, which showed strong silica export [39] but no evidence of enhanced particulate carbon export [7], [40]. In a recent study of organic matter composition within mesoscale eddies [9], the authors describe the major factors influencing POC export within the Canary Islands eddy field. It is suggested that phytoplankton community structure, particularly the dominance of CaCO_3_ organisms over diatoms, efficient ballasting, and subsequent low zooplankton activity are the major factors influencing organic matter export within Canary Islands eddies. To evaluate these factors, we analyzed at 1000 m depth the variability in biogenic opal and calcium carbonate, as well as pyropheophorbide, an indicator of mesozooplankton grazing (Fig. 1d). Our results show a carbonate-dominated region with a low percentage of opal, indicative of low silica supply from the nutrient source waters (North Atlantic Central Waters, NACW), as stated by Ragueneu et al., [41]. However, considering the low opal% in this area, its 6-fold increase during the late-winter bloom (Fig. 1d) must indicate important changes in the food web structure. Indeed, associated with this increase in opal there was a decrease in CaCO_3_% and an increase in pyro mole% (Fig. 1d). These data are suggestive of surface silica enrichment because of winter mixing (see deeper mixed layer depth, MLD; Fig. 1a), relative enhancement of diatoms during the early stages of the phytoplankton bloom, and subsequent increase in POC export at 290 m (Fig. 1a).

**Figure 3:**
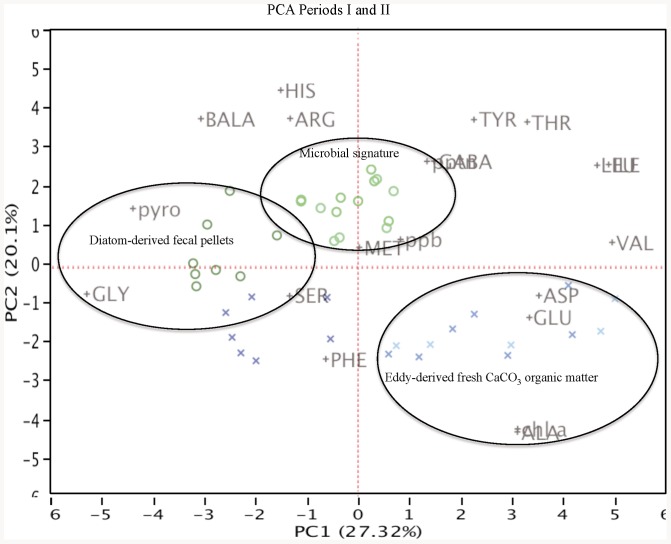
Principal Component Analysis (PCA). PCA was used here to quantitatively assess variation in the organic composition of eddy- vs. bloom-derived sinking particles. PC1 (which explains 27.3% of the variation) split samples into three major groups: 1) stratified period particles, with fresh and CaCO_3_ algal indicators aspartic (ASP) and glutamic (GLU) acids and Chl-*a* located to the right along PC1, 2) bloom-derived particles, indicated by alteration products such as serine (SER), glycine (GLY) and phyropheophorbide (pyro), which are plotted towards the left on PC1, and 3) particles enriched in microbial degradation indicators β-alanine (BALA), γ-aminobutyric acid (GABA), and pheophytin (ppt). Period I (crosses), Period II (circles). Gradually color from light to dark indicates depth levels (290, 500 and 1000 m).

Surprisingly, the signal of the POC peak generated during the seasonal bloom is missed at 500 m (Fig. 1b). This raises the question: what is the fate of the organic carbon exported during the late-winter bloom? The increase in mole% pyro at 1000 m suggests a high contribution of organic matter processed by mesozooplankton (fecal pellets). This hypothesis is supported by direct microscopic observations, which confirm a high proportion of fecal pellets at 1000 m during the seasonal bloom, and by a principal component analysis (PCA) based on pigment and amino acid compositions (Fig. 3). PCA splits the sample set into three major groups and indicates that material collected at 1000 m during the late-winter bloom is enriched in markers typical of diatom-derived fecal pellets. These findings suggest a carbon flux mediated by vertically migrating zooplankton and/or myctophids feeding in surface and upper mesopelagic waters, bypassing the depth of 500 m and defecating in the deep scattering layer (DSL; 600–800 m depth). Since the DSL is particularly well developed and constant in the Canary Island waters [42], defecation by migrant organisms could potentially contribute significantly to the vertical carbon flux below the mesopelagic zone [43]. However, our results show that the POC T_eff_ between 290 and 1000 m during the seasonal bloom was only 8%, but ranged from 27 to 73% in the presence of eddies during the stratified periods I and III (Table 1). Moreover, the PCA based on pigment and amino acid compositions indicates that samples collected during the seasonal bloom at 290 and 500 m presented a stronger microbial signature than other samples (Fig. 3), suggesting that sinking POC at these depths is rapidly processed by the microbial community. Additionally, a fraction of the organic carbon biosynthesized during the seasonal bloom would be bypassed by migrants to the DSL and transformed to non-sinking POC, which is in agreement with the low sinking POC transfer efficiency at 1000 m. In a previous study in this area [44], the authors reported the presence of peaks of dissolved organic carbon (DOC) at 600 m depth, coinciding with the depth of the DSL. Moreover, profiles of suspended POC in the upper 1000 m show peaks, more intense at the DSL, which could only be explained by in situ production [45]. All together, these results suggest that an important fraction of the POC transported down by migrant organisms could be directly (excretion) or indirectly (fecal pellet disaggregation or dissolution) released to the water column as dissolved and suspended organic carbon, decreasing the efficiency of the carbon pump.

**Figure 4:**
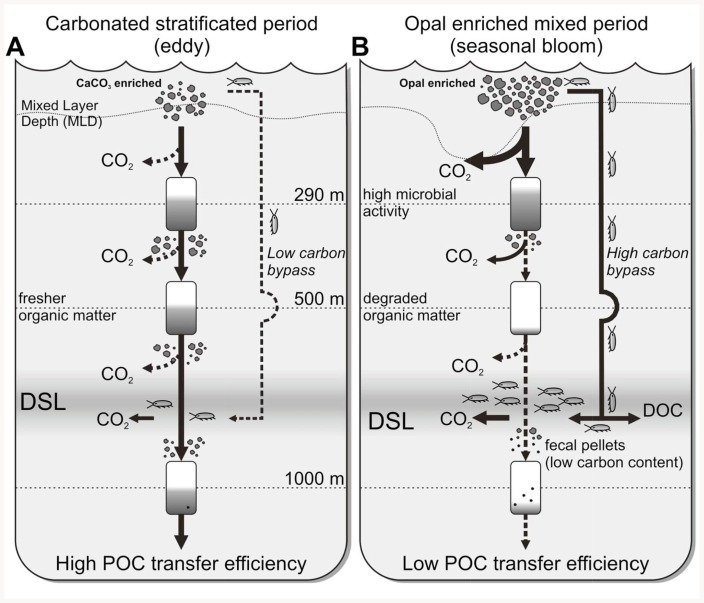
Conceptual model. Conceptual model of POC flow during (A) stratified/eddy conditions and (B) bloom period. A) The POC flux from the epipelagic to the mesopelagic zone is because of passive sedimentation of CaCO_3_-enriched organic aggregates. The low zooplankton and microbial pressure result in a high POC transfer efficiency. B) The POC flow is channeled through active transport mediated by migrant zooplankton. A high microbial and zooplankton activity over the opal-enriched organic matter seems to recycle the exported carbon instead of being transported to the deep ocean, yielding a low POC transfer efficiency (see text for details).

Figure 4 shows a conceptual model of the POC flow during stratified/eddy vs. seasonal bloom conditions based on our observations. The major differences between the two scenarios are the phytoplankton community structure and the resulting differential microbial and zooplankton pressure. The lower metabolic carbon consumption during stratified/eddy conditions (Fig. 4a) could be the cause of the higher POC T_eff_ and the fresher organic matter exported relative to the bloom period (Fig. 4b). Overall, our results suggest that the pathways of POC flow both vary seasonally and at the mesoscale level, with profound implications for carbon dynamics.

Our time series observations provide insights of how variations between CaCO_3_ and opal generating organisms may explain observed differences in carbon export. When opal is more abundant in this region (presumably due to a diatom enrichment during the early stages of the phytoplankton bloom) there is higher carbon export at surface (290 m), probably caused by the generation of larger size cells, but also enhanced grazing by zooplankton and microbial remineralization in the mesopelagic waters, which may result in a lower POC T_eff_ to the deep ocean. The presence of CaCO_3_ enriched samples associated with eddy events during stratified periods could be due to a shallower mixed layer favoring the presence of phytoplankton with carbonate shells against diatoms with silica shells [46]. We did not carry out microscopic analyses of the plankton community composition, and CaCO_3_ in trap samples could also be derived from foraminifera and pteropods, which can be non-trivial constituents of plankton communities in subtropical regions.

### Annual influence of cyclonic eddies on carbon sequestration

**Figure 5:**
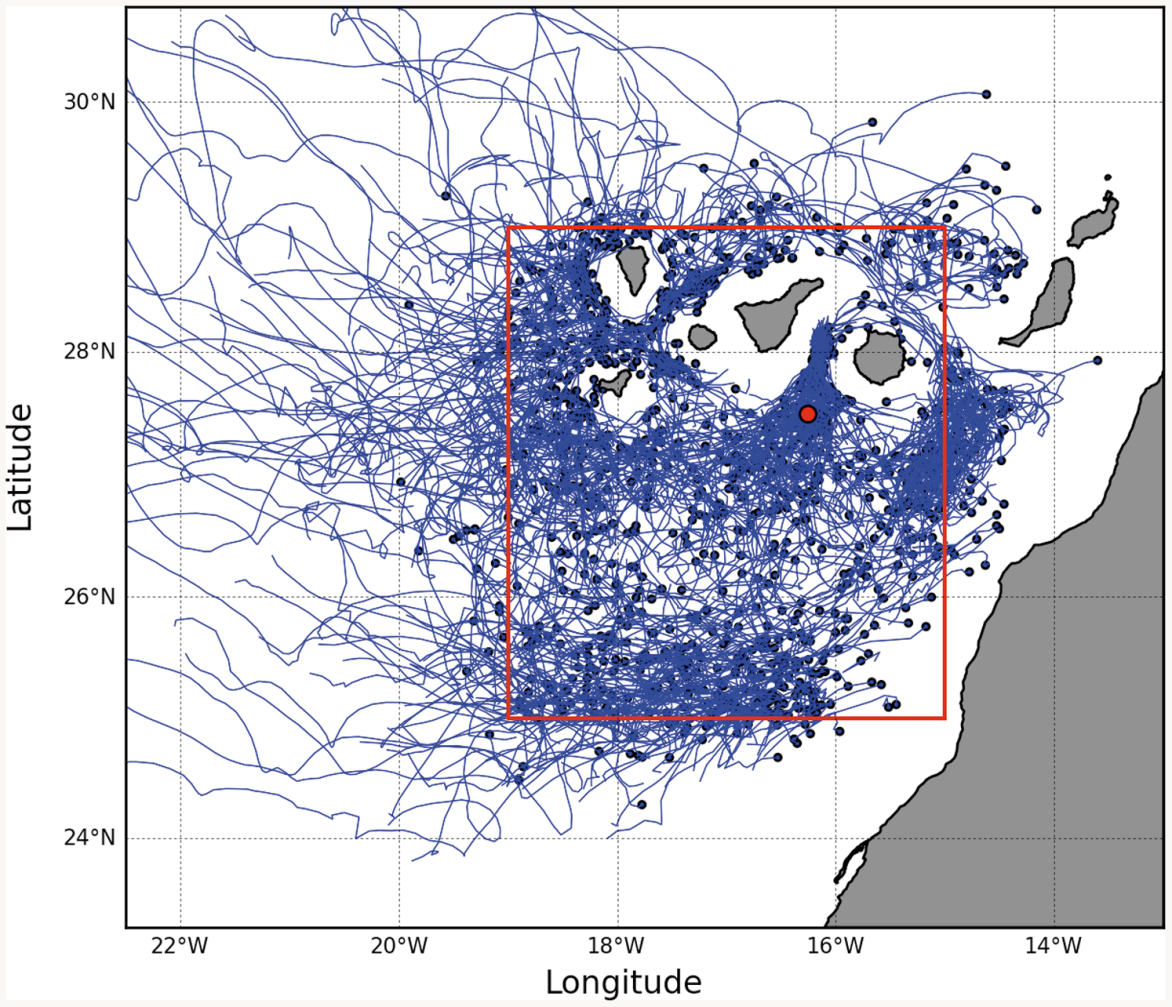
Trajectories of cyclonic eddies. Trajectories of 1160 simulated cyclonic eddies that traverse the Canary Island region (identified by the red box) over a period of 40 climatological years. Blue circles mark the beginning of each trajectory. The large red circle marks the site of the mooring.

**Figure 6:**
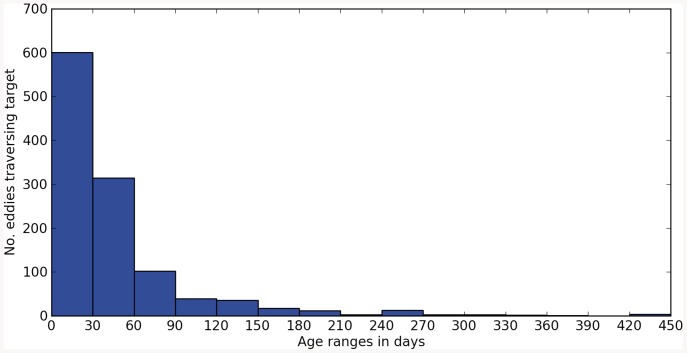
Age of eddies traversing target. Histogram showing the relationship between the frequency at which simulated cyclonic eddies pass through the target region and their age. 30-day bins are shown, and eddies older than 450 days are omitted.

**Figure 7:**
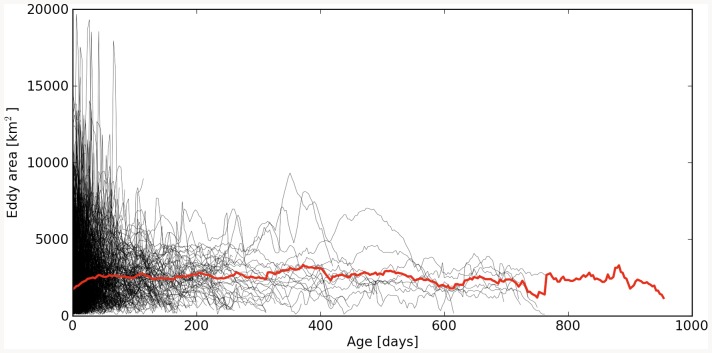
Eddies area. Line plots in black showing the areas of the 1160 simulated cyclonic eddies as a function of their age. The average area is plotted in red.

To evaluate the potential role of cyclonic eddies in the regional carbon budget, we have estimated potential annual eddy-induced carbon export below 1000 m depth by combining different tools. In situ observations and results from the ROMS simulation were used to estimate the number, age and area of cyclonic eddies generated during the stratified summer-autumn period (when eddies seem to enhance carbon fluxes). The ROMS model showed 1160 cyclonic eddies within the 40-year climatological dataset, giving an average of 29 cyclonic eddies per summer-autumn period for the whole area (Fig. 5). Figure 6 shows the relationship between the frequency at which simulated cyclonic eddies pass through the target region and their age, while black lines in Figure 7 show the areas of the 1160 simulated cyclonic eddies as a function of their age. As illustrated in Figure 6, the age histogram for cyclonic eddies shows that most of the eddies are structures of less than 150 days (as are our sampled eddies).

To estimate annual carbon export below 1000 m promoted by the presence of cyclonic eddies within this area, we have used the above data together with the average eddy-induced carbon flux increase obtained in this study as follows:
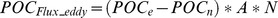
where POC_e_ and POC_n_ are the respective average POC fluxes under eddy and non-eddy conditions (from Table 1), A is the average eddy area, and N is the number of cyclonic eddies generated during summer-autumn within the red box. In our case,
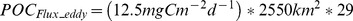



According to these data, the annual carbon export induced by cyclonic eddies is 0.34 Tg C/yr. For comparison, carbon export below 1000 m for the whole studied area without taking into account cyclonic eddies ranges between 0.23–0.36 Tg C/yr when using our non-eddy conditions data or those from Neuer et al., [47] at the ESTOC station. Our results indicate that cyclonic eddies, which represent 28% of the total area, export a similar amount or 1.5 times more carbon than the whole area, clearly enhancing the biological pump. Moreover, our eddy-induced carbon export estimates are likely conservative because sediment traps tend to undercollect particles when deployed in areas of high mesoscale activity [48]–[50].

### Role of cyclonic eddies and migrant zooplankton on the mesopelagic carbon imbalance

Estimates of the plankton metabolic carbon demand can be significantly higher than vertical fluxes of POC measured with sediment traps [1], [2], [51]. We wondered whether intermittent POC pulses by cyclonic eddies could locally resolve this observational discrepancy. Our results indicate that the overall effect of eddy activity in our study area is to increase POC fluxes 2–4 times. To examine the balance between eddy-induced vertical POC fluxes and mesopelagic carbon demand, we used lower and upper thresholds (9 and 68 mmol C m^−2^ d^−1^) for mesopelagic respiration rates in our region of study [52], [53]. Using these respiration rates, our eddy-induced POC fluxes range between 10–50% of the mesopelagic respiration estimates. Thus, we find that cyclonic eddies cannot directly bridge the gap between vertical carbon fluxes and the metabolic carbon demand in mesopelagic waters. This result indicates the existence of alternative mechanisms to fulfill the high carbon demand of mesopelagic waters. Indeed, Baltar et al., [2] found a significant correlation between suspended POC (POC_susp_) and potential respiration in the deep waters of the subtropical Northeast Atlantic. Nevertheless, POC_susp_ concentrations at depth appear to be inadequate to support sustained metabolic demand since a new supply of POC_susp_ would be required to keep up with the demand [1]. Recently, Alonso-González el al., [45] showed that the lateral flux of POC_susp_ from the continental margin accounted for up to 60% of the total mesopelagic respiration in the Canary region, giving evidence of an important mechanism supplying POC_susp_ at deep levels. In addition, Baltar et al., [54] suggested that dissolved inorganic carbon fixation in the dark ocean could contribute between 12–72% to the prokaryotic carbon demand.

Here, we propose a new source of non-sinking organic carbon at depth that may represent a seasonally important fraction of the missing carbon respired in the mesopelagic waters. As stated above, the organic matter produced during phytoplankton blooms could be processed by migrant organisms and released daily as DOC or POC_susp_ in the DSL. Thus, diel vertical migration by zooplankton and myctophids in this area is more likely to supply organic carbon for respiration in the mesopelagic zone, rather than to sequester it to the deep ocean (>1000 m).

### Conclusions

Our study provides evidence that cyclonic eddies generated south of Gran Canaria enhance organic carbon, amino acid and pigment export with respect to non-eddy conditions, even during the seasonal phytoplankton bloom. The higher POC T_eff_ observed during eddy conditions together with the fresher organic matter exported make eddies an efficient organic carbon pump to the ocean interior. The fact that the organic matter exported within eddies is less degraded indicates a faster particle settling velocity (due to differences in particle size or ballasting) or physical protection. This finding has profound implications for carbon sequestration since the depth of organic matter decomposition determines whether respired CO_2_ may be exchanged quickly with the atmosphere or rather be sequestered over long periods of time [55]. Thus, if fast-sinking particles contribute largely to the carbon flux within cyclonic eddies, the POC transfer efficiency to the mesopelagic waters increases, resulting in an enhanced CO_2_ sequestration in the deep ocean (>1000 m). However, we estimated that the highest POC fluxes observed in this study could explain only about 50% of the lowest mesopelagic respiration rates reported for this area. Thus, the apparent metabolic imbalance in the mesopelagic waters of the Canary Island region cannot be satisfied by eddy-derived vertical inputs of sinking POC, strengthening the current view that microbial life in the deep ocean is also dependent on other sources of carbon.

## References

[1] Steinberg DK, Van Mooy BAS, Buesseler KO, Boyd PW, Kobari T, et al (2008) Microbial vs. zooplankton control of sinking particle flux in the ocean's twilight zone. Limnol Oceanogr 53: 1327–1338.

[2] Baltar F, Arístegui J, Gasol JM, Sintes E, Herndl GJ (2009) Evidence of prokaryotic metabolism on suspended particulate organic matter in the dark waters of the subtropical North Atlantic. Limnol and Oceanogr 54: 182–193.

[3] Gruber N, Lachkar Z, Hartmut F, Marchesiello P, Münnich M, et al (2011) Eddy-induced reduction of biological production in eastern boundary upwelling systems. Nature Geosci 4: 787–792.

[4] Sweeney EN, McGillicuddy DJ, Buesseler KO (2003) Biogeochemical impacts due to mesoscale eddy activity in the Sargasso Sea as measured at the Bermuda Atlantic Time Series Study (BATS). Deep-Sea Res II 50: 3017–3039.

[5] Bidigare RR, Benitez-Nelson C, Leonard CL, Quay PD, Parsons ML, et al (2003) Influence of a cyclonic eddy on microheterotroph biomass and carbon export in the lee of Hawaii. Geophys Res Lett 30: 1318 doi:10.1029/2002GL016393

[6] McGillicuddy DJ, Anderson LA, Bates NR, Bibby T, Buesseler KO, et al (2007) Eddy/Wind Interactions Stimulate Extraordinary Mid-Ocean Plankton Blooms. Science 316: 1021–1026. 17510363 10.1126/science.1136256

[7] Benitez-Nelson CR, McGillicuddy DJ (2008) Mesoscale physical-biological-biogeochemical linkages in the open ocean: an introduction to the results of the E-Flux and EDDIES Programs. Deep-Sea Res II 55: 1133–38.

[8] Maiti K, Benitez-Nelson C, Rii YM, Bidigare RR (2008) Influence of a mature cyclonic eddy on particulate export in the lee of Hawaii. Deep-Sea Res II 55: 1445–1460.

[9] Alonso-González IJ, Arístegui J, Lee C, Calafat A (2010a) Regional and temporal variability of sinking organic matter in the subtropical northeast Atlantic Ocean: a biomarker diagnosis. Biogeosciences 7: 2101–2115 doi:10.5194/bg-7-2101-2010

[10] Sangrà P, Auladell M, Marrero-Díaz A, Pelegrí JL, Fraile-Nuez E, et al (2007) On the nature of oceanic eddies shed by the Island of Gran Canaria. Deep-Sea Res I 54: 687–709.

[11] Jiménez B, Sangrà P, Mason E (2008) A numerical study of the relative importance of wind and topographic forcing on oceanic eddy shedding by tall deep water islands. Ocean Modelling 22: 146–157 doi: 10.1016/j.ocemod.2008.02.004

[12] Piedeleu M, Sangrá P, Sánchez-Vidal A, Fabrés J, Gordo C, et al (2009) An observational study of oceanic eddy generation mechanisms by tall deep-water islands (Gran Canaria). Geophys Res Lett 36: L14605 doi:10.1029/2008GL037010

[13] Heussner S, Ratti C, Carbonne J (1990) The PPS 3 time-series sediment trap and the trap sample processing techniques used during the ECOMARGE experiment. Cont Shelf Res 10: 943–958.

[14] Sangrà P, Pelegrí JL, Hernández-Guerra A, Arregui I, Martín JM, et al (2005) Life history of an anticyclone eddy. J Geophys Res 110: C03021 doi:10.1029/2004JC002526

[15] Sangrà P, Pascual A, Rodríguez-Santana A, Machín F, Mason E, et al (2009) The Canary Eddy Corridor: A major pathway for long-lived eddies in the subtropical North Atlantic. Deep Sea Res I 56: 2100–2114.

[16] UNESCO (1994) Protocols for the Joint Global Ocean Flux Study (JGOFS) Core Measurement, Intergovernmental Oceanographic Commission. Manual and Guides 29: 169.

[17] Turnewitsch R, Springer BM, Kiriakoulakis K, Vilas JC, Arístegui, et al (2007) Determination of particulate organic carbon (POC) in seawater: The relative methodological importance of artificial gains and losses in two glass-fiber-filter-based techniques. Mar Chem 105: 208–228.

[18] Lee C, Wakeham SG, Hedges JI (2000) Composition and flux of particulate amino acids and chloropigments in equatorial Pacific seawater and sediments. Deep-Sea Res I 47: 1535–1568.

[19] Wakeham SG, Lee C, Peterson ML, Liu Z, Szlosek J, et al (2009) Organic biomarkers in the twilight zone - Time series and settling velocity sediment traps during MEDFLUX. Deep-Sea Res II doi:10.1016/j.dsr2.2008.11.030

[20] Dauwe B, Middelburg J (1998) Amino acids and hexosamines as indicators of organic matter degradation state in North Sea sediments. Limnol Oceanogr 43: 782–798.

[21] Ingalls AE, Lee C, Wakeham SG, Hedges JI (2003) The role of biominerals in the sinking flux and preservation of amino acids in the Southern Ocean along 170°W. Deep-Sea Res II 50: 713–738.

[22] Goñi MA, Yunker MB, Macdonald RW, Eglinton TI (2000) Distribution and sources of organic biomarkers in Arctic sediments from the Mackenzie River and Beaufort shelf. Mar Chem 71: 23–51.

[23] Ingalls AE, Liu Z, Lee C (2006) Seasonal trends in the pigment and amino acid compositions of sinking particles in biogenic CaCO3 and SiO2 dominated regions of the Pacific sector of the Southern Ocean along 170°W. Deep-Sea Res I 53: 836–859.

[24] Goutx M, Wakeham S, Lee C, Duflos M, Guigue C, et al (2007) Composition and degradation of marine particles with different settling velocities in the northwest Mediterranean Sea. Limnol Oceanogr 52: 1645–1664.

[25] Sheridan CC, Lee C, Wakeham SG, Bishop JKB (2002) Suspended particle organic composition and cycling in surface and midwaters of the equatorial Pacific Ocean. Deep-Sea Res I 49: 1983–2008.

[26] Shchepetkin AF, McWilliams JC (2009) Correction and Commentary for “Ocean Forecasting in Terrain-Following Coordinates: Formulation and Skill Assessment of the Regional Ocean Modeling System” by Haidvogel et al. J Comp Phys 227: 3595–3624. J Comput Phys 228: 8985–9000.

[27] Chelton DB, Schlax MG, Samelson RM, de Szoeke RA (2007) Global observations of large oceanic eddies. Geophys Res Lett 34: L15606.

[28] Mason E (2009) High-resolution modelling of the Canary Basin oceanic circulation. University of Las Palmas de Gran Canaria. Thesis, 259 p.

[29] Mason E, Colas F, Molemaker J, Shchepetkin AF, Troupin C, et al (2011) Seasonal variability of the Canary Current: A numerical study. J Geophys Res 116: C06001 doi:10.1029/2010JC006665

[30] Chelton DB, Schlax MG, Samelson RM (2011) Global observations of nonlinear mesoscale eddies. Prog Oceanogr 91: 167–216.

[31] Mason E, Pascual A, McWilliams JC (2013) A new sea surface height based code for oceanic mesoscale eddy tracking. J Atmos Oceanic Technol Submitted manuscript

[32] Arístegui J, Sangrá P, Hemández-León S, Cantón M, Hernández-Guerra A, et al (1994) Island-induced eddies in the Canary Islands. Deep-Sea Res 41: 1509–1525.

[33] Arístegui J, Tett P, Hernández-Guerra A, Basterretxea G, Montero MF, et al (1997) The influence of island-generated eddies on chlorophyll distribution: a study of mesoscale variation around Gran Canaria. Deep-Sea Res I 44: 71–96.

[34] Barton ED, Arístegui J, Tett P, Cantón M, García-Braun J, et al (1998) The transition zone of the Canary Current upwelling region. Progress in Oceanography 41: 455–504.

[35] Basterretxea G, Arístegui J (2000) Mesoscale variability in phytoplankton biomass distribution and photosynthetic parameters in the Canary – NW African coast transition zone. Marine Ecology Progress Series 197: 27–40.

[36] Williams RG (2011) Ocean eddies and plankton blooms. Nature Geos 4: 739–740.

[37] Yu EF, Francois R, Bacon MP, Honjo S, Fleer AP, et al (2001) Trapping efficiency of bottom-tethered sediment traps estimated from the intercepted fluxes of 230Th and 231Pa. Deep Sea Res I 48: 865–889.

[38] Scholten JC, Fietzke J, Vogler S, Rutgers van der Loeff MM, Mangini A, et al (2001) Trapping efficiencies of sediment traps from the deep Eastern North Atlantic: The 230th calibration. Deep Sea Res II 48: 2383–2408.

[39] Benitez-Nelson C, Bidigare R, Dickey TD, Landry MR, Leonard CL, et al (2007) Mesoscale eddies drive increased silica export in the subtropical Pacific Ocean. Science 316: 1017–1021. 17510362 10.1126/science.1136221

[40] Buesseler KO, Lamborg C, Cai P, Escoube R, Johnson R, et al (2008) Particle fluxes associated with mesoscale eddies in the Sargasso Sea. Deep Sea Res II 55: 1426–1444.

[41] Ragueneau O, Tréguer P, Leynaert A, Anderson RF, Brzezinski MA, et al (2000) A review of the Si cycle in the modern ocean: recent progress and missing gaps in the application of biogenic opal as a paleoproductivity proxy. Global Planet Change 26: 317–365.

[42] Hernández-León S, Gómez M, Pagazaurtundua M, Portillo-Hahnefeld A, Montero I, et al (2001) Vertical distribution of zooplankton in Canary Island waters: implications for export flux. Deep-Sea Res I 48: 1071–1092.

[43] Hernández-León S, Franchy G, Moyano M, Menéndez I, Schmoker C, et al (2010) Carbon sequestration and zooplankton lunar cycles: Could we be missing a major component of the biological pump? Limnol Oceanogr 55 (6) 2503–2512.

[44] Arístegui J, Barton ED, Montero MF, García-Muñoz M, Escánez J (2003) Organic carbon distribution and water column respiration in the NW Africa-Canaries Coastal Transition Zone. Aquat Microb Ecol 33: 289–301.

[45] Alonso-González IJ, Arístegui J, Vilas JC, Hernández-Guerra A (2009) Lateral POC transport and consumption in surface and deep waters of the Canary Current region: a box model study. Global Biogeochem Cy 23: GB2007 doi:10.1029/2008GB003185

[46] Cermeño P, Dutkiewicz S, Harris RP, Follows M, Schofield O, et al (2008) The role of nutricline depth in regulating the ocean carbon cycle. Proc Natl Acad Sci, USA 105: 20344–20349. 19075222 10.1073/pnas.0811302106 PMC2603260

[47] Neuer S, Cianca A, Helmke P, Freudenthal T, Davenport R, et al (2007) Biogeochemistry and hydrography in the eastern subtropical North Atlantic gyre, Results from European time-series station ESTOC. Prog Oceanogr 72: 1–29.

[48] Butman CA (1986) Sediment trap biases in turbulent flows: results from a laboratory flume study. Journal of Marine Research 44: 645–693.

[49] Gust G, Bowles W, Giordano S, Hüttel M (1992) Particle accumulation in a cylindrical sediment trap under laminar and turbulent steady flow: an experimental approach. Aquatic Sciences 58: 297–326.

[50] Alonso-González IJ, Arístegui J, Lee C, Sánchez-Vidal A, Calafat A, et al (2010b) Role of slowly settling particles in the ocean carbon cycle. Geophys Res Lett 37: L13608 doi:10.1029/2010GL043827

[51] Burd AB, Hansell DA, Steinberg DK, Anderson TR, Arístegui J, et al (2010) Assessing the apparent imbalance between geochemical and biochemical indicators of meso- and bathypelagic biological activity: what the @$#! is wrong with present calculations of carbon budgets? Deep Sea Res 57 (16) 1557–1571.

[52] Jenkins WJ, Goldman JC (1985) Seasonal oxygen cycling and primary production in the Sargasso Sea. J Mar Res 43: 465–491.

[53] Arístegui J, Duarte CM, Gasol JM, Alonso-Sáez L (2005) Active mesopelagic prokaryotes support high respiration in the subtropical northeast Atlantic Ocean. Geophys Res Lett 32: L03608 doi:03610.01029/02004GL021863

[54] Baltar F, Arístegui J, Sintes E, Gasol JM, Reinthaler T, et al (2010) Significance of non-sinking particulate organic carbon and dark CO_2_ fixation to heterotrophic carbon demand in the mesopelagic northeast Atlantic. Geophys Res Lett 37: L09602 doi:10.1029/2010GL043105

[55] Armstrong RA, Lee C, Hedges JI, Honjo S, Wakeham SG (2002) A new mechanistic model for organic carbon fluxes in the ocean: based on the quantitative association of POC with ballast minerals. Deep-Sea Res II 49: 219–236.

